# Depletion of C3orf1/TIMMDC1 Inhibits Migration and Proliferation in 95D Lung Carcinoma Cells

**DOI:** 10.3390/ijms151120555

**Published:** 2014-11-10

**Authors:** Huiling Wu, Wenbing Wang, Huaxi Xu

**Affiliations:** 1 School of Food and Biological Engineering, Jiangsu University, Zhenjiang 212013, China; E-Mail: huiling@ujs.edu.cn; 2 Institute of Life Sciences, Jiangsu University, Zhenjiang 212013, China; 3 School of Medical Science and Laboratory Medicine, Jiangsu University, Zhenjiang 212013, China; E-Mail: wenbingwang@ujs.edu.cn

**Keywords:** C3orf1 (TIMMDC1), mitochondria, microarray, lung carcinoma, proliferation, migration

## Abstract

In our previous study, we identified an association of high expression of *c3orf1*, also known as TIMMDC1 (translocase of inner mitochondrial membrane domain-containing protein 1), with metastatic characteristics in lung carcinoma cells. To investigate the preliminary function and mechanism of this mitochondrial protein, we depleted C3orf1 expression by introducing siRNA into 95D lung carcinoma cells. We demonstrated that C3orf1 depletion significantly suppressed 95D cell growth and migration. We confirmed C3orf1 localization in the inner mitochondrial membrane and showed that mitochondrial viability, membrane potential, and ATPase activity were remarkably reduced upon depletion of C3orf1. Microarray data indicated that genes involved in regulation of cell death, migration, and cell-cycle arrest were significantly altered after C3orf1 depletion for 48 h. The expression of genes involved in focal adhesion, ECM-receptor interaction, and p53-signaling pathways were notably altered. Furthermore, cell-cycle arrest genes such as CCNG2 and PTEN as well as genes involved in cell migration inhibition, such as TIMP3 and COL3A1, were upregulated after C3orf1 depletion in 95D cells. Concurrently, expression of the migration-promoting gene NUPR1 was markedly reduced, as confirmed by real-time PCR. We conclude that C3orf1 is critical for mitochondrial function, migration, and proliferation in 95D lung carcinoma cells. Depletion of C3orf1 inhibited cell migration and cell proliferation in association with upregulation of genes involved in cell-cycle arrest and cell migration inhibition. These results suggest that C3orf1 (TIMMDC1) may be a viable treatment target for lung carcinoma, and that further study of the role of this protein in lung carcinoma pathogenesis is justified.

## 1. Introduction

Lung carcinoma ranks among the most common cancers in the world and is one of the leading causes of cancer-related death. The American Cancer Society estimates that 224,210 new lung and bronchus cancer cases and 159,260 deaths will occur in the United States in 2014 [1]. In China, there were a total of 605,946 lung cancer cases and 486,555 deaths in 2010, according to the annual report of cancer statistics. The incidence of lung cancer was the highest among that of other clinical cancers [2]. Lung cancer is classified as small cell lung cancer (SCLC) or non-small cell lung cancer (NSCLC), which is the most common type, accounting for 85%–90% of all lung cancer cases [3].

The carcinogenesis and progression of lung cancer has an etiology that includes multiple steps and genes. Conventional treatment, including surgery, radiotherapy, and chemotherapy, has greatly improved, and a number of new biological therapies such as gene therapy and immune therapy have emerged. However, treatment efficacy remains unsatisfactory [4]. Most NSCLC tumors develop slowly. They are generally discovered in the late stages and grow and metastasize rapidly. Conventional radiotherapy is able to control only 30% of unresectable NSCLCs. The two-year survival rate with conventional radiotherapy is 40%. Outcomes for NSCLC patients remain poor, with overall cure rates of less than 20% [5]. Thus, novel diagnostic and therapeutic approaches are needed. Challenges in improving NSCLC outcome include integration of rapid advances in clinical, pathological, and molecular knowledge of the disease [6,7]. Proliferation, invasion, and metastasis have increasingly been the focus of research in recent years [7,8]. The invasiveness of malignant lung carcinoma contributes greatly to treatment challenges.

In our previous study, the human *c3orf1* gene, also known as TIMMDC1 (translocase of inner mitochondrial membrane domain-containing protein 1), was identified as being overexpressedin 95D lung carcinoma cells with highly metastatic characteristics by using differential display PCR (ddPCR) [9]. Bioinformatic analysis showed that the *c3orf1* gene, which is located on chromosome 3p13.33, is predicted to encode a mitochondrial membrane transport protein, as determined using MITOPROT online software. The probability of mitochondrial targeting by C3orf1, as determined using this software, is 0.9271. Recently, it was found that C3orf1 participated in constructing the membrane arm of mitochondrial complex I [10]. It was further reported that C3orf1 is a membrane-embedded mitochondrial complex I assembly (MCIA) factor, which functions through association with the MCIA complex [11].

It is well known that mitochondrial complex I plays a vital role in coupling electron transfer to the release of protons into the mitochondrial inner membrane space to generate ATP. Studies have been conducted to investigate the correlation of complex I dysfunction with lung cancer [12,13,14,15]. However, the function of the *c3orf1* gene in lung carcinoma cells is unclear. In the present study, we introduced *c3orf1* siRNA into 95D NSCLC cells to provide additional evidence for the function and preliminary mechanism of C3orf1 protein in the context of metastatic lung cancer.

## 2. Results

### 2.1. C3orf1 Gene Expression Is Higherin 95D Cells than in 95C Lung Carcinoma Cells

**Figure 1:**
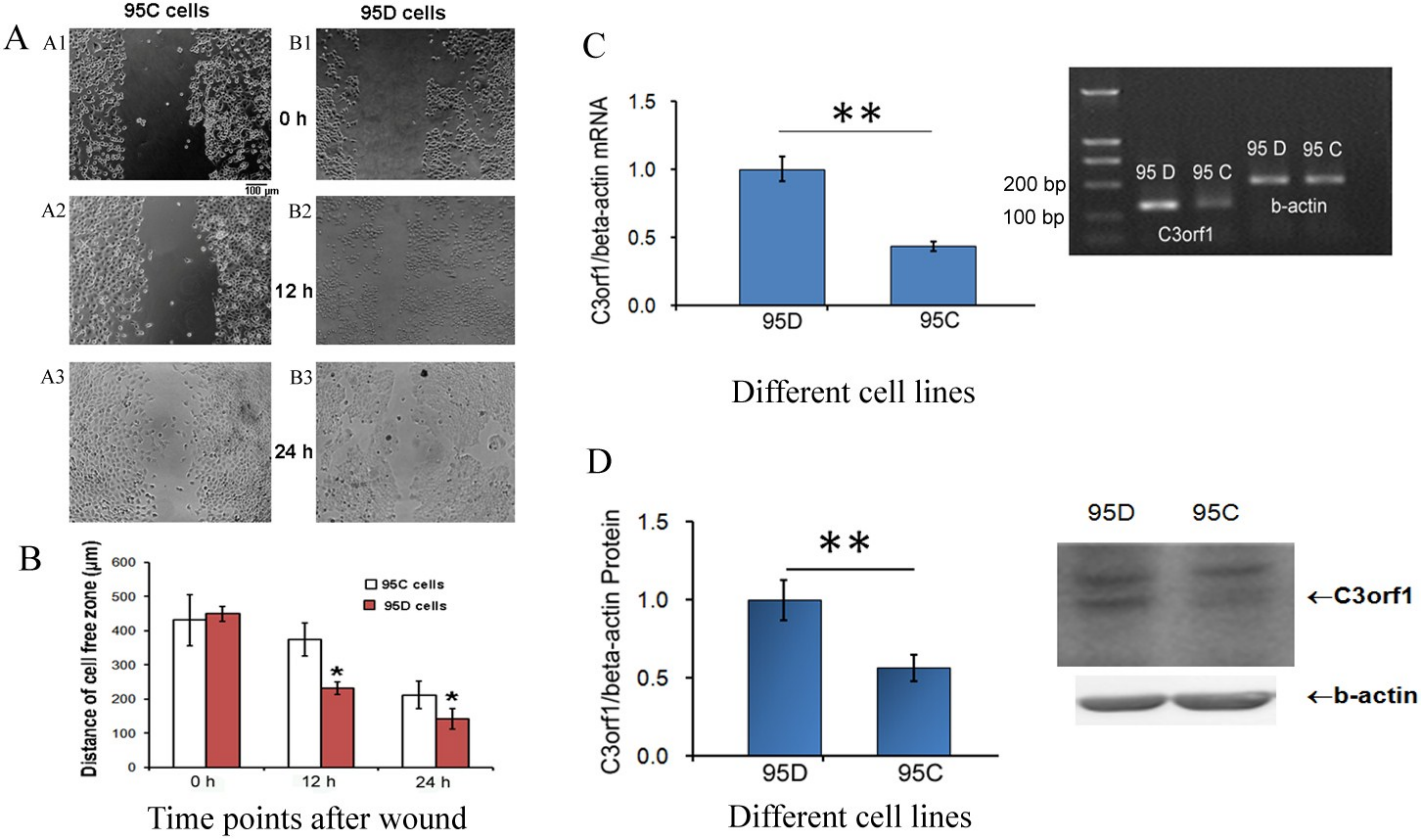
Expression of the C3orf1 gene is high in migratory 95D lung carcinoma cells. (**A**) Results of wound-healing assays in 95C and 95D cells. Panel A1–A3, representative images of scratch-wounded 95C cells at 0, 12, and 24 h. Panel B1–B3, representative images of scratch-wounded 95D cells at 0, 12, and 24 h. Scale bar = 100 µm; (**B**) Statistical results of wound healing assays in 95C and 95D cells. Measurements of the distance of wounded cell-free space at the indicated time points. Data are represented as mean ± SD; *n =* 3; * *p <* 0.05; (**C**) Results of real-time quantitative PCR measuring the relative *c3orf1* mRNA level in lung carcinoma 95D and 95C cells. β-actin was used as an internal control. Data are represented as mean ± SD. The data were normalized (*i.e.*, the average expression level of *c3orf1* in 95D cells was set to 1, and the average expression level of *c3orf1* in 95C cells was calculated in relation to this averaged value); *n =* 4, ** *p* < 0.01. The insert panel is a representative image of the semi-quantitative RT-PCR gel; and (**D**) Results of Western blotting analysis used to detect the relative C3orf1 protein level in lung carcinoma 95D and 95C cells. β-actin was used as the internal control. Data are represented as mean ± SD. The data were normalized as described in Figure 1C; *n =* 3, ** *p* < 0.01. The insert panel shows representative images of Western blotting results.

Previously, we used ddPCR to identify a high level of *c3orf1* mRNA in 95D cells with metastatic characteristics compared to that in AGS (gastric carcinoma cells), MGC-803 (gastric carcinoma cells), LTEP (lung adenocarcinoma cells), TE1 (esophageal carcinoma cells), and U937 (macrophages) cells. 95C and 95D cells are derived from NSCLC, but have different metastasis-related characteristics [9]. In the present study, we determined the migration ability and C3orf1 gene expression in 95D and 95C cells. To determine the rates of migration in these cell lines, scratch-wound assays were conducted. At 0, 12, and 24 h after wounding, wound widths in 95C cells were 431.3 ± 75.6, 375.0 ± 47.6, and 212.5 ± 39.6 µm, respectively. In 95D cells, wound widths were 450.0 ± 21.5, 231.3 ± 18.5, and 141.7 ± 29.7 µm, respectively. As shown in Figure 1A,B, wounded 95D cells migrated 35.4 µm more than 95C cells after 24 h (212.5/2 and 141.7/2, respectively; *p <* 0.05). Differences in C3orf1 gene expression between 95D and 95C cells were detected using real-time PCR and Western blotting. Results of this analysis indicated that C3orf1 mRNA and protein were 2.32 (Figure 1C) and 1.77-fold (Figure 1D) higher in 95D cells than those in 95C cells, respectively (*p <* 0.01).

### 2.2. Depletion of c3orf1 Inhibits Cell Proliferation and Migration in 95D Cells

**Figure 2:**
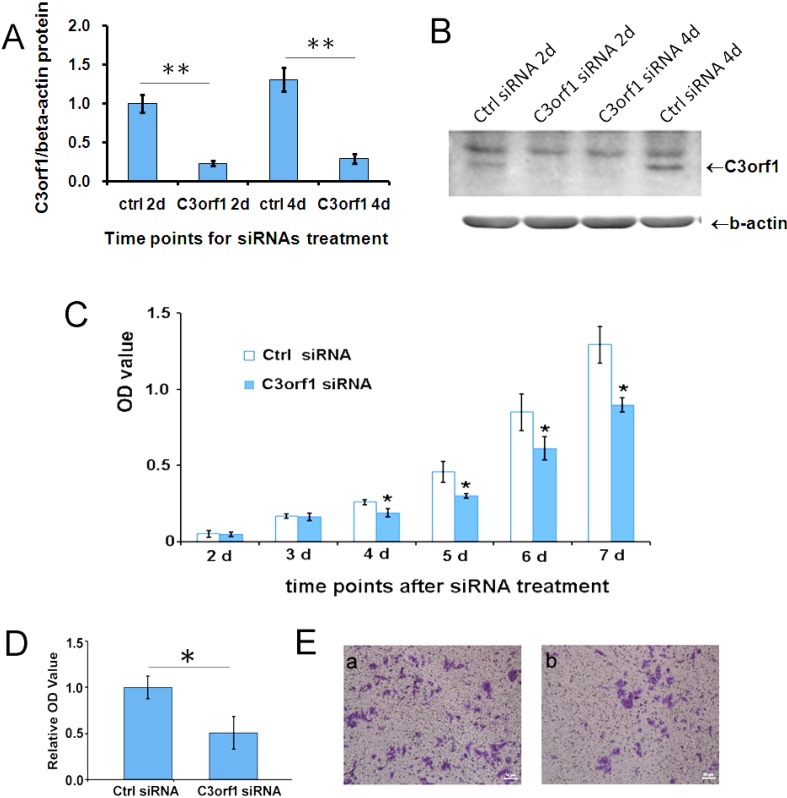
Depletion of C3orf1 in 95D cells inhibits cell proliferation and migration. (**A**) The efficiency of different siRNAs was evaluated by Western blotting after siRNA transfection in 95D cells for 2 days (2d) and 4 days (4d). β-actin was used as an internal control. Data are represented as mean ± SD. The data were normalized as described above, and the average expression level of C3orf1 protein in 95D cells treated with ctrl siRNA for 2 days (2d) was set to 1; ** *p* < 0.01, *n =* 3; (**B**) Representative images of Western blotting results; (**C**) The cell proliferation curve indicating that depleting C3orf1 expression suppressed the proliferation of 95D cells significantly between day 4 (4d) and day 7 (7d). The data are represented as mean ± SD; * *p <* 0.05, *n =* 3; (**D**) Trans-well assays indicating that depleting C3orf1 expression decreased cell migration. Data are represented as mean ± SD. The data were normalized, and the average number of migrated 95D cells treated with ctrl siRNA was set to 1; * *p <* 0.05, *n =* 4; and (**E**) Representative images of stained trans-well assay membranes. Scale bar = 50 µm.

To ascertain the cellular effects of C3orf1, we utilized siRNA to deplete C3orf1 in 95D cells. As shown in Figure 2A,B, 77% and 78% of the C3orf1 protein was depleted from 95D cells after *c3orf1* siRNA treatment for 2 days and 4 days, respectively (*p <* 0.01). The proliferation of 95D cells with or without *c3orf1* siRNA treatment was detected using a CCK8 kit (Dojindo Co., Kumamoto, Japan). Results of this assay are shown in Figure 2C. The depletion of C3orf1 significantly suppressed 95D cell growth (*p <* 0.05). A marked decline in cell growth began on the fourth day of culture. In the trans-well assays after cell migration for an additional 18 h, the number of migrated 95D cells was reduced by 49.4% upon C3orf1 depletion compared to that observed with control siRNA treatment (Figure 2D; *p <* 0.05). These results demonstrated that targeting C3orf1 represses cell proliferation and migration of 95D lung carcinoma cells.

### 2.3. C3orf1 Localizes to the Inner Mitochondrial Membrane of 95D Cells and Exhibits Mitochondria-Related Functions

**Figure 3:**
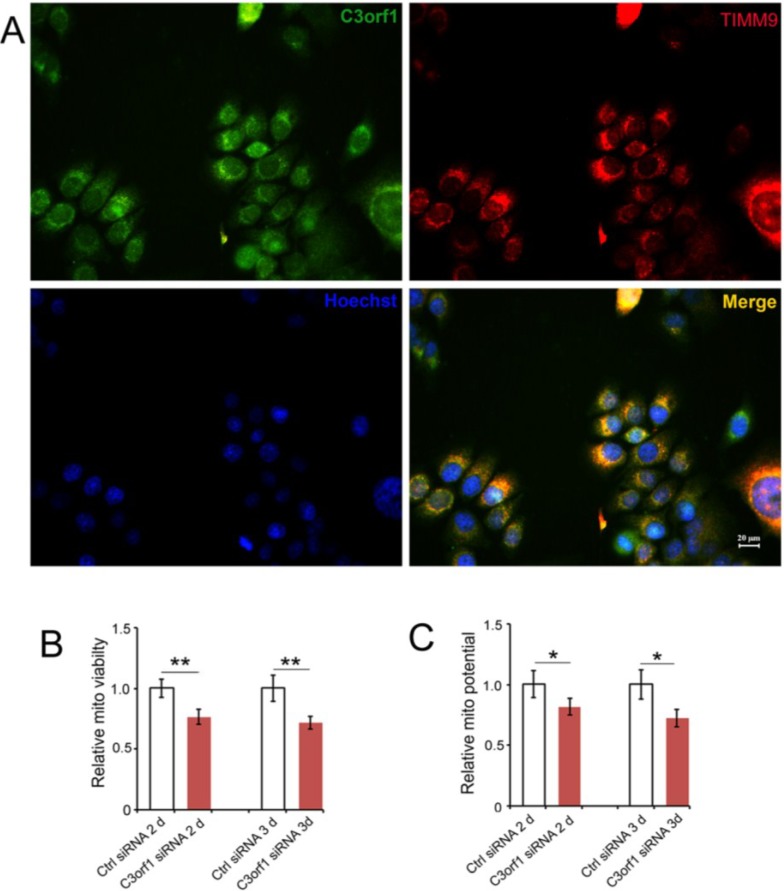

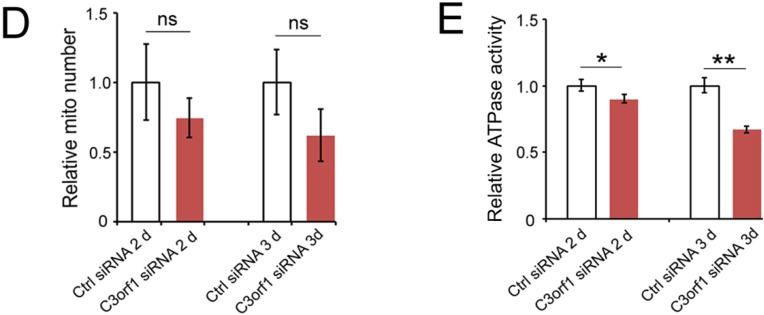
C3orf1 localizes to the inner mitochondrial membrane of 95D cells and C3orf1 knockdown affects mitochondria-related functions; (**A**) Representative images of immunostaining with anti-C3orf1 antibody (in green) and anti-Timm9 antibody (in red). The nucleus was labeled with Hoechst33342 dye (in blue). Scales bar = 20 µm; (**B**) Results of mitochondrial viability assays on 95D cells subjected to control or *c3orf1* siRNA treatment for 2 days (2d) and 3 days (3d). Data are represented as mean ± SD. The data were normalized as described above, and the average mitochondrial viability of 95D cells treated with ctrl siRNA was set to 1; ** *p <* 0.01, *n =* 3; (**C**) Results of mitochondrial potential assays on 95D cells subjected to control or *c3orf1* siRNA treatment for 2 days (2d) and 3 days (3d). Data are represented as mean ± SD. The data were normalized, and the average mitochondrial potential of 95D cells treated with ctrl siRNA was set to 1; * *p <* 0.05, *n =* 3; (**D**) Results of mitochondrial number assays in 95D cells subjected to control or *c3orf1* siRNA treatment for 2 days (2d) and 3 days (3d). Data are represented as mean ± SD. The data were normalized, and the average mitochondrial number of 95D cells treated with ctrl siRNA was set to 1; *n =* 3; ns = no significant change in mitochondrial numbers between cells treated with control siRNA or *c3orf1* siRNA; and (**E**) Results of ATPase activity assays on 95D cells subjected to control or *c3orf1* siRNA treatment for 2 days (2d) and 3 days (3d). Data are represented as mean ± SD. The data were normalized, and the average ATPase activity of 95D cells treated with ctrl siRNA was set to 1; * *p* < 0.05, ** *p <* 0.01, *n =* 3.

We used the online bioinformatic software MITOPROT to determine that C3orf1 has a probability of 0.9271 for being a mitochondrial membrane transport protein. Therefore, we also investigated the localization of C3orf1 protein in 95D cells using immunostaining with antibodies that bind C3orf1 and TIMM9, which is an inner mitochondrial membrane marker. TIMM9 co-localized with C3orf1 protein (Figure 3A). We then further investigated the effect of C3orf1 depletion on mitochondrial viability, number of mitochondria, mitochondrial membrane potential, and ATPase activity in 95D cells. As shown in Figure 3B,C, mitochondrial viability and the membrane potential were significantly decreased upon C3orf1 depletion by 23.4% and 18.4% at 2 day, and 28.3% and 27.8% at 3 day (*p <* 0.01 and 0.05), respectively. However, there was no significant change in the number of mitochondria in 95D cells upon *c3orf1* siRNA treatment. In addition, mitochondrial ATPase activity was measured to investigate the effect of C3orf1 depletion on mitochondrial ATP generation in 95D cells. The result shown in Figure 3E demonstrates that the ATPase activity in C3orf1 depleted 95D cells was reduced by 10.1% at 2 day and 23.3% at 3 day compared to that in control cells (*p <* 0.05 and 0.01), respectively. Therefore, we concluded that C3orf1 protein knockdown significantly affected mitochondrial functions in 95D lung carcinoma cells.

### 2.4. C3orf1 Depletion Resulted in Alteration of Gene Expression Profiles in 95D Cells

**Figure 4:**
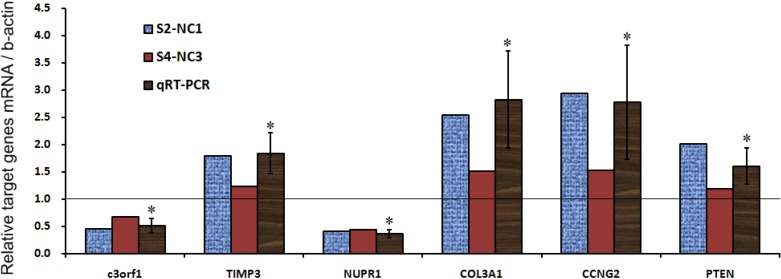
Alteration in the gene expression profile of C3orf1-depleted cells. Graphic representation of real-time PCR results depicting the expression of selected genes; * p < 0.05.

To investigate the molecular signature of C3orf1 depletion in 95D cells, Affymetrix Genechip Prime View Human Gene Expression Array was used according to the method described in a previous study [16]. Cells treated with *c3orf1* or control siRNA were screened for differentially expressed genes. We identified 439 upregulated probe sets corresponding to 360 unique genes and open reading frames and 98 downregulated probe sets corresponding to 61 genes and open reading frames (fold change > 2) in the *c3orf1* siRNA treated cells compared to the control siRNA treated cells. Using gene ontology (GO) analysis (Table 1), these genes were identified as being involved in several biological processes related to tumor suppression, such as regulation of apoptosis, programmed cell death, cell-cycle arrest, and regulation of cell migration. With Kyoto Encyclopedia of Genes and Genomes (KEGG) pathway enrichment, several of the altered pathways that were most likely to be involved in tumor invasion were identified, including focal adhesion and ECM-receptor interaction pathways (Table 2). Interestingly, the p53 signaling pathway, which is associated with cell proliferation, is involved. Based on these results, we chose transcripts of interest, such as TIMP3, NUPR1, COL3A1, CCNG2, and PTEN, for confirmation of array results by using real-time PCR. As shown in Figure 4 and Table 3, the cell-cycle arrest-related transcripts CCNG2 [17] and PTEN [18] were significantly upregulated in *c3orf1* siRNA-treated cells compared to control cells (*p <* 0.05). Transcripts involved in cell migration inhibition, TIMP3 [19] and COL3A1 [20], were upregulated. The NUPR1 transcript [21], a migration promoting gene, was markedly downregulated upon C3orf1 depletion in 95D cells.

**Table 1:** Genes of GO functional category analysis that were differentially expressed in 95D cells after *c3orf1* siRNA treatment for 48 h (*p* < 0.05).

GO TERM_BP_FAT	Count	%	*p* Value
0042981~regulation of apoptosis	44	11.028	3.47 × 10^−7^
0043067~regulation of programmed cell death	44	11.028	4.54 × 10^−7^
0010941~regulation of cell death	44	11.028	4.97 × 10^−7^
0016125~sterol metabolic process	13	3.258	4.24 × 10^−6^
0043065~positive regulation of apoptosis	26	6.516	2.98 × 10^−5^
0043068~positive regulation of programmed cell death	26	6.516	3.34 × 10^−5^
0016044~membrane organization	24	6.015	3.46 × 10^−5^
0010942~positive regulation of cell death	26	6.516	3.60 × 10^−5^
0008203~cholesterol metabolic process	11	2.757	5.86 × 10^−5^
0010627~regulation of protein kinase cascade	18	4.511	8.52 × 10^−5^
0010033~response to organic substance	35	8.772	8.58 × 10^−5^
0008202~steroid metabolic process	16	4.010	8.70 × 10^−5^
0010035~response to inorganic substance	16	4.010	1.02 × 10^−4^
0016126~sterol biosynthetic process	7	1.754	1.47 × 10^−4^
0008219~cell death	34	8.521	1.80 × 10^−4^
0016265~death	34	8.521	2.02 × 10^−4^
0001666~response to hypoxia	12	3.008	3.21 × 10^−4^
0009628~response to abiotic stimulus	21	5.263	4.51 × 10^−4^
0043405~regulation of MAP kinase activity	12	3.008	4.96 × 10^−4^
0070482~response to oxygen levels	12	3.008	4.96 × 10^−4^
0048545~response to steroid hormone stimulus	14	3.509	6.19 × 10^−4^
0043066~negative regulation of apoptosis	20	5.013	7.27 × 10^−4^
0031667~response to nutrient levels	14	3.509	7.88 × 10^−4^
0001932~regulation of protein amino acid phosphorylation	13	3.258	8.03 × 10^−4^
0032268~regulation of cellular protein metabolic process	24	6.015	8.47 × 10^−4^
0043069~negative regulation of programmed cell death	20	5.013	8.57 × 10^−4^
0060548~negative regulation of cell death	20	5.013	8.89 × 10^−4^
0006915~apoptosis	27	6.767	0.00210
0009991~response to extracellular stimulus	14	3.509	0.00214
0030334~regulation of cell migration	12	3.008	0.00218
0005996~monosaccharide metabolic process	14	3.509	0.00233
0012501~programmed cell death	27	6.767	0.00261
0007050~cell-cycle arrest	9	2.256	0.00293
0006006~glucose metabolic process	11	2.757	0.00332
0045859~regulation of protein kinase activity	18	4.511	0.00334
0043549~regulation of kinase activity	18	4.511	0.00470
0001568~blood vessel development	14	3.509	0.00534
0040012~regulation of locomotion	12	3.008	0.00578
0051270~regulation of cell motion	12	3.008	0.00598
0051896~regulation of protein kinase B signaling cascade	4	1.003	0.00685
0002685~regulation of leukocyte migration	4	1.003	0.01090
0030198~extracellular matrix organization	8	2.005	0.01131
0051726~regulation of cell cycle	16	4.010	0.01188
0000187~activation of MAPK activity	7	1.754	0.01265
0022402~cell-cycle process	23	5.764	0.01430
0051241~negative regulation of multicellular organismal process	10	2.506	0.01550
0010629~negative regulation of gene expression	21	5.263	0.01579
0045860~positive regulation of protein kinase activity	12	3.008	0.01652
0051347~positive regulation of transferase activity	12	3.008	0.02669
0051247~positive regulation of protein metabolic process	12	3.008	0.02892
0051674~localization of cell	14	3.509	0.02983
0048870~cell motility	14	3.509	0.02983
0016477~cell migration	13	3.258	0.03028
0042127~regulation of cell proliferation	28	7.018	0.03137
0006928~cell motion	19	4.762	0.03161
0044265~cellular macromolecule catabolic process	26	6.516	0.03624
0042060~wound healing	10	2.506	0.03677
0008629~induction of apoptosis by intracellular signals	5	1.253	0.03780
0006917~induction of apoptosis	14	3.509	0.03945
0012502~induction of programmed cell death	14	3.509	0.04030
0009611~response to wounding	20	5.013	0.04407
0045787~positive regulation of cell cycle	5	1.253	0.04474
0007568~aging	7	1.754	0.04522
0008285~negative regulation of cell proliferation	15	3.759	0.04650

**Table 2:** Genes of KEGG pathway functional classification that were differentially expressedin 95D cells after *c3orf1* siRNA treatment for 48 h (out of 537 genes, *p <* 0.05).

Term	%	*p* Value	Genes
04144:Endocytosis	3.01	0.016	*VTA1*, *HSPA1A*, *EEA1*, *HSPA1B*, *CLTC*, *CHMP2B*, *TFRC*, *HSPA2*, *CXCR4*, *NEDD4*, *MDM2*, *HSPA8*, *AP2M1*
04510:Focal adhesion	3.01	0.029	*CAV1*, *ROCK2*, *ITGB8*, *ITGAV*, *COL3A1*, *PPP1R12A*, *BIRC3*, *THBS1*, *PPP1CB*, *PTEN*, *COL5A1*, *FN1*
04512:ECM-receptor interaction	1.75	0.032	*ITGB8*, *ITGAV*, *COL3A1*, *THBS1*, *SV2C*, *COL5A1*, *FN1*
04910:Insulin signaling pathway	2.26	0.039	*PPP1R3C*, *IRS2*, *PPP1R3B*, *PRKAG2*, *HK2*, *RPS6KB1*, *RPS6*, *PCK2*, *PPP1CB*
04920:Adipocytokine signaling pathway	1.50	0.042	*IRS2*, *PRKAG2*, *SLC2A1*, *NFKBIA*, *ACSL4*, *PCK2*
04115:p53 signaling pathway	1.50	0.045	*MDM2*, *CCNG1*, *THBS1*, *IGFBP3*, *CCNG2*, *PTEN*

**Table 3:** Relative mRNA expression of target genes in C3orf1-depleted 95D cells compared to control cells.

Genes/Ctrl	*c3orf1*	*TIMP3*	*NUPR1*	*COL3A1*	*CCNG2*	*PTEN*
**S2-NC1**	0.4499	1.7926	0.4058	2.5374	2.9360	2.0069
**S4-NC3**	0.6704	1.2319	0.4367	1.5181	1.5311	1.1950
**qRT-PCR mean**	0.5099	1.8332	0.3613	2.8237	2.7743	1.6045
**SD**	0.1263	0.3748	0.0722	0.8942	1.0438	0.3368
***p* value**	0.0052	0.0183	0.0001	0.0242	0.0349	0.0359

S2-NC1, S4-NC3 = two repeated microarray analysis results comparing *c3orf1* siRNA (S2 or S4) treatment to control siRNA treatment (NC1 or NC3). The qRT-PCR mean, SD, and *p* value represent the average of 3–4 replicates, standard deviation, and Student’s *t*-test probability value, respectively. Numbers of genes/ctrl >1 indicate upregulation. Numbers of genes/ctrl < 1 indicate downregulation.

## 3. Discussion

Human C3orf1 protein was previously predicted to be a mitochondrial membrane transport protein. Recently, this protein was found to serve as an important assembly factor of mitochondrial complex I [11]. It is well known that mitochondria play a vital role in many cellular processes and that the functions of the mitochondria vary with cell type and cell status. The most important mitochondrial function is the production of energy through oxidative phosphorylation, and another is the process of programmed cell death. Mitochondrial complex-I is involved in intra-molecular electron transfer and in mediating reactive oxygen species (ROS) generation [22,23]. Recent studies have illustrated the correlation of complex I disorder with lung cancer [12,13]. The use of ROS as a potent killer of cancer cells has also been investigated [24]. Aspirin, which may non-selectively inhibit COX-1and COX-2, also appears to confer protection against lung cancer [25].

We previously identified the *c3orf1* gene being highly expressed in 95D cells by using ddPCR [9]. In the present study, we provided additional evidence for the function and preliminary mechanism of C3orf1 protein. After determining that 95D cells exhibit a migratory phenotype and highly express C3orf1 mRNA and protein, we depleted C3orf1 by using siRNA. C3orf1 depletion significantly reduced cellular proliferation and migration. We confirmed that C3orf1 protein co-localizes with TIMM9 (translocase of inner mitochondrial membrane 9) [26], a marker of the inner mitochondrial membrane. Furthermore, we determined that C3orf1 depletion markedly decreased mitochondrial viability, membrane potential, and ATPase activity in 95D cells. However, the number of mitochondria in living cells exhibited no significant reduction. This may be explained by that mitochondria have a certain range of redundancy and variability in number, ranging from one to 10,000 and averaging at about 200. In addition, this could also be attributed to treatment with siRNA for a relatively short time.

We utilized microarray as a high-throughput method to identify genes, involved in cellular proliferation and migration, which were regulated upon C3orf1 depletion in 95D cells. Thus, we performed gene ontology analysis by Affymetrix human cDNA microarray. Through the results of gene ontology analysis, KEGG pathway enrichment, and real-time PCR confirmation, we demonstrated that focal adhesion, ECM receptor interaction, p53 signaling, cell-cycle arrest, and migration promotion pathways were regulated upon blockade of C3orf1.

We conclude that depletion of C3orf1 in 95D lung carcinoma cells impacts mitochondrial viability, membrane potential, and ATPase activity, and can inhibit cell migration and proliferation in association with upregulation of cell-cycle arrest genes, and downregulation of migration genes. Our present analysis indicates that the C3orf1 may be considered as a novel candidate target for inhibiting lung carcinoma proliferation and migration. 

## 4. Experimental Section

### 4.1. Cell Culture

The human lung carcinoma cell lines 95C and 95D were kindgifts from Dr. Wenxin Qin at the State Key Laboratory of Oncogenes (Shanghai, China) and were routinely cultured in Dulbecco’s Modified Eagle’s Medium (DMEM; Invitrogen, Carlsbad, CA, USA) supplemented with 10% fetal bovine serum (FBS, GE Healthcare Life Sciences HyClone Laboratories, Logan, UT, USA) and antibiotics (penicillin/streptomycin).

### 4.2. RNA Extraction and Real-Time PCR Analysis

According to the methods followed in our previous study [27], total RNA was isolated from cells by using TRIzol reagent (Life Technologies, Grand Island, NY, USA). The first strand cDNA was synthesized using an Omniscript Reverse Transcription Kit (Qiagen, Venlo, Limburg, The Netherlands) in a 20-μL reaction system containing 2 μg total RNA. A1-μL aliquot of the first-strand cDNA was amplified using primers (listed in Table 4) designed to investigate the expression of *c3orf1* by real-time PCR. The reaction mixtures included 10 µL of 2× Fast Evagreen qPCR Master Mix (Biotium, Hayward, CA, USA), 2 µL of 10× ROX (Biotium), gene specific primers at a final concentration of 0.5 µM, and 1 µL of cDNA. Real-time PCR was performed in a StepOne Real-time PCR system (ABI Applied Biosystems, Grand Island, NY, USA). The thermal cycling program consisted of 2 min at 96 °C, followed by 45 cycles of 15 s at 96 °C and 1 min at 60 °C. Data collection was performed during the 60 °C extension step. To account for variability in the total RNA input, the expression of target genes was normalized to that of the β-actin gene. In addition, a negative control without first-strand cDNA was prepared. The relative expression was calculated using the comparative 2^−Δ*C*t^ method and all data were expressed as the means ± SD. Differences between groups were analyzed by one-way analysis of variance (ANOVA) using SPSS software (IBM, Armonk, NY, USA). Results were considered to be significant at * *p <* 0.05 and ** *p <* 0.01.

**Table 4:** siRNA sequences and sequences of oligonucleotides used for qRT-PCR.

Gene Name	Sequence (5'→3')
*c3orf1* sense	5'-GAGACCTTCAACACCCCAGCC-3'
*c3orf1* antisense	5'-CCTGAACAGTCTCACCACTACTTACTTC-3'
beta-actin sense	5'-GACTACCTCATGAAGATCCTCACC-3'
beta-actin antisense	5'-TCTCCTTAATGTCACGCACGATT-3'
ctrl siRNA sense	5'-UUCUCCGAACGUGUCACGUTT-3'
ctrl siRNA antisense	5'-ACGUGACACGUUCGGAGAATT-3'
*c3orf1* siRNA sense	5'-UGUAGAGCAUUGUGCCUAUTT-3'
*c3orf1* siRNA antisense	5'-AUAGGCACAAUGCUCUACATT-3'
CCNG2 sense	5'-TTTGGATCGTTTCAAGGCGC-3'
CCNG2 antisense	5'-TTGATCACTGGGAGGAGAGC-3'
PTEN sense	5'-TGCAGTATAGAGCGTGCAGA-3'
PTEN antisense	5'-CTGGATTTGACGGCTCCTCT-3'
COL3A1 sense	5'-GGCAAAGATGGAACCAGTGG-3'
COL3A1 antisense	5'-TCACCTCCAATCCCAGCAAT-3'
TIMP3 sense	5'-CTGTGCAACTTCGTGGAGAG-3'
TIMP3 antisense	5'-AGTGTTTGGACTGGTAGCCA-3'
NUPR1 sense	5'-CTGACCTCTATAGCCTGGCC-3'
NUPR1 antisense	5'-GGTCACCAGTTTCCTCTCGT-3'

### 4.3. Wound-Healing Assay

Cell migration was evaluated using the scratch wound assay according to our previous experiments [16]. The 95C and 95D cells were cultured on 24-well plates for 2 days to form a tight cell monolayer and were wounded with a 200-µL plastic pipette tip. The remaining cells were washed twice with culture medium to remove cell debris and incubated at 37 °C. At the indicated times, migrating cells at the wound front were photographed and the width of the wound area relative to that at the 0 h time point was measured using Image-Pro Plus version 6.2 software (Media Cybernetics, Rockville, MD, USA).

### 4.4. siRNA Treatment and Western Blotting

The sequences for siRNAs used in this study were designed and synthesized by Biomics Biotechnologies Inc. (Nantong, China). The sequences of the universal control (Ctrl) siRNA and gene-specific *c3orf1* siRNA are listed in the Table 4. Protocols used for siRNA experiments were based on our previous study [27]. For studies involving siRNA in cultured cells, isolated and dissociated cells were transfected using Lipofectamine2000 (Life Technologies, Grand Island, NY, USA) according to the manufacturer’s instructions. After transfection, cells were re-plated on 35-mm culture dishes. After culture for 2 and 4 days to permit protein depletion, the cells were collected for Western blotting analysis. The cells were homogenized in a cell lysis buffer. Total protein was quantified using BCA analysis. Proteins were separated using SDS-PAGE. After transfer to a PVDF membrane (Millipore, Bedford, MA, USA), the membrane was blocked with 5% non-fat dry milk in Tris-buffered saline (TBS, pH 7.4) and was incubated overnight with antibodies against C3orf1 (Santa Cruz Biotechnology, Dallas, TX, USA; 1:2500) or β-actin (Sigma-Aldrich, St. Louis, MO, USA; 1:7500) at 4 °C. After washing with TBS/T (TBS with 0.1% Tween 20), IRDye 800-conjugated affinity purified donkey anti-rabbit IgG (Rockland Immunochemicals Inc., Gilbertsville, PA, USA; 1:5000) was applied at room temperature for 1 h. The images were scanned with an Odyssey infrared imaging system (LI-COR, Lincoln, NE, USA), and the data were analyzed with PDQuest 7.2.0 software (Bio-Rad, Hercules, CA, USA). For loading normalization and relative quantitative analysis of C3orf1 expression, β-actin was used as an internal control.

### 4.5. Determination of Cell Proliferation Activity and Migration Assay

To measure cell proliferation, 95D cells were transfected with control or *c3orf1* siRNA for 24 h, expanded in culture, trypsinized, and plated onto 96-well plates at equal densities. Beginning the following day, a Cell Counting Kit-8 (CCK-8, Dojindo Laboratories, Kumamoto, Japan) was used to examine cell proliferation once daily for 6 days, according to the manufacturer’s protocol and previous studies [28,29]. Briefly, 10 μL of CCK-8 solution was added into each well and incubated for 2 h, after which the absorbance at 450 nm was measured using a microplate reader (BioTek, Winooski, VT, USA). Measurements were repeated at least three times. The migration of control or *c3orf1* siRNA-transfected 95D cells was examined using 6.5-mm trans-well chambers with 8-μm pores (Costar, Cambridge, MA, USA). One hundred microliters of medium containing 3 × 10^4^ suspended cells (that were already treated with *c3orf1* siRNA for 2 days) was transferred to the top chambers of each trans-well. Six hundred microliters of complete medium was added into the lower cell-free chambers. After allowing migration to occur for 18 h, the non-migrated cells on the upper surface of each membrane were removed with a cotton swab. Cells adhering to the bottom surface of each membrane were stained with 0.1% crystal violet, imaged, and counted using a DMR inverted microscope (Leica Microsystems, Wetzlar, Germany). Assays were performed in triplicate wells for each time point.

### 4.6. Immunocytochemistry

For investigating C3orf1 localization, the inner mitochondrial membrane marker TIMM9 was chosen as a target for immunostaining. The cultures were incubated with rabbit polyclonal anti-rat TIMM9 antibody (1:200) at 4 °C for 14–16 h and then incubated with the corresponding secondary Cy3-conjugated goat anti-rabbit IgG antibodies at 1:800 (Jackson Immuno Research, West Grove, PA, USA) for 2 h at room temperature. Fluorescence images were obtained on a DMR fluorescence microscope (Leica Microsystems, Wetzlar, Germany).

### 4.7. Measurements of Mitochondrial Viability, Number, Membrane Potential, and ATPase Activity

Probability of C3orf1 being a mitochondrial membrane transport protein was determined using the online bioinformatic software MITOPROT .We used the Mitochondrial Viability Assay reagent (Abcam, Cambridge, England, Cat. ab129732), Mito Tracker Green FM reagent (M&C Gene Technology, Beijing, China, Cat. NC-M7514), and tetramethylrhodamine methyl ester perchlorate (TMRM, Biotium; Cat. 70017) reagents to measure the mitochondrial viability, number, and membrane potential, respectively, according to the manufacturer’s protocols and previous studies [30]. Briefly, 95D cells transfected with control or *c3orf1* siRNA for 24 h were expanded in culture, trypsinized, and plated on 96-well plates at equal densities. At the end of the treatment period, cells were overlaid with 100 μL of 2× mitochondrial viability stain in each well containing 100 μL of medium and incubated under sterile conditions at 37 °C for 4 h. Absorbance at 570 nm was measured using a microplate reader (BioTek, Winooski, VT, USA). For detecting changes in mitochondrial number, Mito Tracker Green FM reagent was diluted to 40 nM with the growth medium. Medium was removed from the wells containing 95D cells, and fresh growth medium containing the dye was added. After incubation for 15 min, the absorbance was measured at 560 nm/590 nm by using the plate reader. For mitochondrial potential detection, cells were incubated in a TMRM (40 nM) solution for 15–20 min at 37 °C in the dark. Absorbance at 570 nm was measured using the plate reader. Mitochondrial ATPase activity was detected as follows. Mitochondria of 95D cells were isolated using the animal cells/tissues mitochondria extraction kit (Genmed Scientifics, Shanghai, China; Cat. GSM10006). Ten micrograms of mitochondria were used to detect ATP synthase activity by using the mitochondrial complex V activity quantitative analysis kit (Genmed Scientifics, Cat. GMS50083). All procedures followed the manufacturer’s instructions. The ATPase activity of 95D cells transfected with control or *c3orf1* siRNA was obtained by measuring absorbance at 340 nm. All measurements were repeated at least three times.

### 4.8. Microarray Analysis

After 95D cells were transfected with TIMMDC1 siRNA or control siRNA for 48 h, 1 mL ofTRIzol reagent (Life Technologies, Grand Island, NY, USA) was added to each culture flask, and the mixture was incubated at 4 °C. Total RNA from the control or TIMMDC1 siRNAs-treated samples were extracted, cleaned up, reverse-transcribed, and hybridized to the Genechip Prime View Human Gene Expression Array (Affymetrix, Santa Clara, CA, USA) by the Gene Company (Nanjing, China). In Affymetrix Gene Chip Command Console Software, the CEL files were preprocessed using the robust multichip average (RMA) method. Gene expression was normalized to the mean expression of the control sample, and detection cells were used to filter for probe sets that were marginally present (in one-fourth of the arrays). Fold-change values for each experimental sample were exported to Excel for mean and SD calculations. Canonical pathways were analyzed using the Ingenuity Pathway Analysis (IPA) software package (Qiagen, Venlo, Limburg, The Netherlands). Genes that met the fold change cutoff of 2 and were associated with a canonical pathway were used for further analysis. Biological processes were analyzed using the PANTHER database [31]. Genes that met the fold change cutoff of 2 and were associated with a biological process with a known GO identification number were considered for analysis.

### 4.9. Statistical Analysis

Comparisons between different experimental groups were performed using repeated measures ANOVA with GraphPad Prism 5.0 software (GraphPad Software Inc., La Jolla, CA, USA). The data were expressed as mean ± SD and analyzed using one-way ANOVA with the SPSS software package (IBM, Armonk, NY, USA). Values of *p <* 0.05 were considered statistically significant.

## 5. Conclusions

In summary, we identified higher expression of C3orf1/TIMMDC1 in 95D lung carcinoma cells and confirmed C3orf1 locals in the inner mitochondrial membrane. We demonstrated that C3orf1 depletion significantly suppressed 95D cell growth and migration. The mitochondrial function related assays showed depletion of C3orf1 remarkably impacted mitochondrial viability, membrane potential, and ATPase activity. Microarray data indicated that genes involved in regulation of cell death, migration, and cell-cycle arrest were significantly altered after C3orf1 depletion for 48 h. Our study indicated that the C3orf1 may be considered as a novel candidate target for inhibiting lung carcinoma proliferation and migration.
